# Amelioration of *Listeria monocytogenes* infection in mice by live and pasteurized *Faecalibacterium prausnitzii* and its cell-free supernatant and its association with strengthened gut barrier and downregulated TLR4/NF-κB pathway

**DOI:** 10.1007/s12602-026-11041-7

**Published:** 2026-05-16

**Authors:** Ailin Wang, Yue Teng, Jinhui Jia, Yunqi Gu, Jian Guo, Xiaodong Xia

**Affiliations:** 1 State Key Laboratory of Marine Food Processing and Safety Control, National Engineering Research Center of Seafood, School of Food Science and Technology, Dalian Polytechnic University, Dalian, Liaoning 116034 China; 2 Hainan Academy of Medical Sciences, Hainan Medical University, Haikou, Hainan 571199 China; 3 Department of Food Science and Nutrition, The Hong Kong Polytechnic University, Hom Hung, Kowloon, Hong Kong China

**Keywords:** *Faecalibacterium prausnitzii*, *Listeria monocytogenes*, Infection, Intestinal barrier function, Inflammatory responses

## Abstract

*Listeria monocytogenes* is a common foodborne pathogen that poses a serious health risk. *Faecalibacterium prausnitzii* is one of the major members of the gut microbiota and is considered essential for maintaining gut health. The aim of this study was to investigate the effect of live *F. prausnitzii* (FP), pasteurized *F. prausnitzii* (pFP) and its cell-free supernatant (CFS) on the susceptibility of mice to *L. monocytogenes* infection and to explore the underlying mechanisms. Safety evaluation results indicated that FP, pFP, and CFS showed no toxic effects in healthy mice. In *L. monocytogenes*-infected mice, these treatments significantly decreased bacterial counts in various organs and feces, and alleviated inflammation. Furthermore, the interventions significantly upregulated short-chain fatty acid (SCFA) levels, particularly acetate and propionate, and modulated the gut microbiota composition after infection. In addition, RT-qPCR and western blot results showed that they reduced inflammatory factors and intestinal damage, which is associated with downregulation of the TLR4/NF-κB pathway. Taken together, these results suggest that FP, pFP and CFS attenuate *L. monocytogenes* infection in mice and could be potentially developed as an alternate strategy for prevention or mitigation of *L. monocytogenes*.

## Highlights

FP, pFP and CFS reduced *L. monocytogenes* infection in mice

FP, pFP and CFS enhanced intestinal epithelial barrier

FP, pFP and CFS modulated the composition of the gut microbiota

FP, pFP and CFS attenuated inflammation by TLR4/NF-κB pathway

## Introduction

*Listeria monocytogenes*is a foodborne pathogen that represents a significant threat to public health [17]. The bacterium can enter the human body through the consumption of contaminated food and is particularly harmful to vulnerable populations, including pregnant women, newborns, the elderly, and immunocompromised individuals. Upon infection, *L. monocytogenes*can cause fever, muscle pain, headaches, nausea, vomiting, and diarrhoea [32]. Listeriosis is a serious disease caused by the *L. monocytogenes*, with a global mortality rate of 20–30%. Recent studies have shown that, although the prevalence of *L. monocytogenes *infection has generally stabilized over the past 10 years, small outbreaks of this more serious and aggressive disease are occurring more frequently than previously thought [41]. In severe cases, the bacterium can invade the central nervous system, leading to meningitis, encephalitis, septicaemia, and even death. The relationship between *L. monocytogenes *infections and the intestinal barrier is complex and the bacterium employs various mechanisms to penetrate and disrupt the intestinal barrier thereby facilitating infection [8]. Additionally, the bacterium secretes proteins, such as Listeria adhesion protein (LAP), which disrupt tight junctions between intestinal epithelial cells, thereby compromising the integrity of the intestinal barrier [8]. The invasion and proliferation of *L. monocytogenes *trigger an inflammatory response in the gut which can exacerbate intestinal barrier damage thereby facilitating further bacterial invasion and dissemination [25]. *L. monocytogenes* infection poses a substantial threat to both the gut and overall human health. An effective vaccine for *L. monocytogenes*is lacking, and patients presenting with severe listeriosis infections are usually treated with β-lactam antibiotics and gentamicin but relies on the immune system for clearance. Immunocompromised individuals are at higher risk of severe, fatal infections [40]. Given the relatively high mortality rate of systemic *L. monocytogenes* infections and the continued emergence of drug-resistant strains, it is critical to explore new safe and effective strategies to prevent listeriosis.

The gut microbiota comprises a diverse group of microorganisms that reside in the human intestinal tract playing multifaceted roles in maintaining intestinal health. Gut microbiota is involved in food digestion and nutrient metabolism assisting the body in absorbing indigestible plant fibers and synthesizing certain vitamins [2]. Recent advances employing integrative multi-omics approaches have provided deeper insights into these interactions. For instance, a study using microbiome, transcriptome, and metabolome analyses demonstrated that *Lactobacillus acidophilus* could modulate gut microbiota composition, host immune responses, and metabolic pathways during *Salmonella Typhimurium*infection. Such findings highlight that the protective mechanisms of probiotics often involve complex multi-layered modulation of the host-microbe ecosystem rather than a single mode of action [18, 39]. Additionally, gut microbiota regulates the balance of the intestinal immune system, influencing immune cell development and function, and reducing the incidence of allergies and inflammatory diseases [30]. Meanwhile, gut microbiota is closely associated with the host’s metabolic and cardiovascular health, influencing body weight and cholesterol levels by regulating energy balance and lipid metabolism [54]. The most important is that intestinal microorganisms defend against pathogenic invasion through competitive exclusion and the production of antimicrobial substances, thereby maintaining the integrity of the intestinal barrier [59]. Yan et al., verified that the berberine-treated intestinal microbiota was sufficient to alleviate the occurrence of colonic tumors associated with colitis in mice [53]. It has also been shown that quercetin increases SCFA levels and decreases pro-inflammatory cytokine levels by modulating intestinal microbiota [10]. Therefore, gut microbes play a crucial role in maintaining gut health and overall physiological function.

*Faecalibacterium prausnitzii*is a Gram-positive, anaerobic enteric bacterium belonging to the Clostridia class within the phylum Firmicutes and is considered a key factor in maintaining intestinal health [21]. It also possesses anti-inflammatory properties that modulate the host’s immune response, thereby reducing intestinal inflammation [24]. Gao et al., showed that *F. prausnitzii *ameliorated ICI-induced colitis, remodeled gut microbial composition, and enhanced the antitumor activity of immunotherapy [12]. Studies have shown that *F. prausnitzii *helps maintain the integrity of the intestinal barrier, preventing pathogens and harmful substances from entering the body through the gastrointestinal tract [56]. More importantly, *F. prausnitzii* regulates immune system balance and reduces the inflammatory response thus regulating the balance of intestinal microbiota and realizing the role of maintaining a stable and healthy intestinal environment. Although *F. prausnitzii* has been extensively examined for its role in chronic disorders such as obesity, diabetes, and cancer, the effects of *F. prausnitzii* (particularly pasteurized *F. prausnitzii* and its cell-free supernatant) on infection with foodborne pathogens have been rarely explored. Therefore, in this study, live *F. prausnitzii* (FP), pasteurized *F. prausnitzii* (pFP), and cell-free supernatant (CFS) were examined for detailed investigation of *L. monocytogenes*, validate that FP, pFP and CFS reduce intestinal epithelial damage, protect intestinal infection, and its mechanism were also explored.

## Materials and methods

### *L. monocytogenes* and *F. prausnitzii*

*L. monocytogenes *10403s was purchased from Eastmo Biotech Co., Ltd. (Beijing, China). The strain was cultured overnight at 37°C before use in Brain-Heart Infusion Broth. *F. prausnitzii *(DSM 17677) was cultured anaerobically in Modified Reinforced Clostridial Broth (MRC broth) at 37°C for 24 h. pFP and CFS were prepared as previously described (J. Liu, [28]). Grown *F. prausnitzii* was centrifuged at 12,000 g for 10 min to remove media, rinsed twice and suspended in 0.01 M PBS, and pasteurized for 30 min at 70°C to get pFP. After centrifugation, the upper layer of liquid was taken and filtered through a 0.22 μm filter (Millipore filter, USA) to obtain CFS of *F. prausnitzii.*

### Animal treatment and research design

All experimental procedures were performed according to the Guide for Laboratory Animals of the National Institute of Health. The study was approved by the Animal Ethics Committee of Dalian Polytechnic University (DLPU2024035). Five-week-old C57BL/6J male mice of specific pathogen-free (SPF) status were purchased from Liaoning Chang Sheng Biotechnology Co., Ltd. (Changchun, China). The mice were housed in a controlled SPF environment (12-hour light/dark cycle, 24 ± 1°C). The mice were divided into five groups (*n* = 10): control group (phosphate-buffered saline), Mod group (phosphate-buffered saline, *L. monocytogenes *in day 21), FP group (1 × 10⁹ CFU/mL), pFP group (1 × 10⁹ CFU/mL), and CFS group (1 × 10⁹ CFU/mL) [3, 11, 45]. After 21 days of FP, pFP, and CFS interventions, the mice were gavaged with 100 µL of CaCO_3_ (suspended in 0.5% CMC-Na) at 150 mg/mL for the first 24 h to neutralize gastric acid and facilitate the survival of *L. monocytogenes *during infection. Subsequently, the mice were infected with 5 × 10⁹ CFU/mL of *L. monocytogenes.* Body weight was recorded daily over a 24-day period. On day 25, the animals were euthanized following body weight measurements. Blood was collected by centrifugation at 1,000 × g for 10 min at 4°C. Serum was isolated and stored at −80°C for future analysis. Colonic samples were collected and stored at −80°C. Colon samples were fixed in 4% paraformaldehyde for 24 h. Animals were randomly assigned to experimental groups based on body weight after acclimatization. Sample collection was performed in an unblinded manner due to the nature of the interventions, whereas key outcome assessments, such as bacterial enumeration and histopathological scoring, were performed by personnel blinded to the group assignments. 

### HE staining

Colon tissues were fixed in 4% paraformaldehyde and then subjected to a longitudinal sectioning procedure with three samples per group [46]. The samples were subsequently embedded in paraffin blocks. The tissues were sectioned at 3–5 μm thickness using a microtome, followed by staining with hematoxylin and eosin (HE). Images were captured using a Nikon Eclipse Ti-S microscope (Tokyo, Japan). The degree of submucosal oedema, epithelial integrity, goblet cell retention, and cellular infiltration of the lamina propria were evaluated in each tissue section under the microscope, with scores ranging from 0 to 4 for each criterion. The pathological score is the sum of the scores from these four criteria. Inflammation was classified into four categories: severe inflammation (score ≥ 12), moderate inflammation (score 8–12), mild inflammation (score 4–8), and no inflammation (score ≤ 4) [28].

### AB-PAS staining

AB-PAS staining was performed following the manufacturer’s instructions. After euthanizing the mice colon tissues were collected and paraffin sections were prepared [44]. The sections were dehydrated, stained with a drop of Alisin Blue (AB) for 15 min, washed with deionized water, and dried. They were then oxidized with periodic acid for 5 min, washed with deionized water, and immersed in Schiff reagent for 5 min, followed by differentiation with Acid Differentiation Solution for 5 s. Following washing and drying with deionized water, the sections were differentiated with Scott Blue Solution for 5 min. After washing and drying with deionized water, the sections were re-blued with Scott’s blueing solution for 5 min, then dehydrated and sealed. Finally, the slides were observed using a Nikon microscope (Tokyo, Japan).

### Enumeration of *L. monocytogenes* in feces and organs

Mouse tissues (ileum, colon, intestinal lymph nodes, liver, and spleen) and feces were aseptically collected and homogenized in cold PBS containing 1% Triton X-100 (1:10, w/v). Serial dilutions were prepared and incubated at 37°C for 24 h, followed by inoculation onto *Listeria*Chromogenic Medium plates (HB7008, Hopebio, Qingdao, China) to determine the total colony count [45].

### ELISA analysis

The levels of cytokines were measured by ELISA Kits according to the manufacturer’s instructions, including IL-6, IL-1β, IL-10, TNF-α, DAO, LPS and IFN-γ (Jiancheng, Nanjing, China).

### Western blotting

RIPA lysis buffer (Beyotime) supplemented with protease and phosphatase inhibitors was used to extract proteins from colon tissues subjected to various treatments [29]. Equal amounts (30 mg) of protein samples were separated by 10–15% SDS-polyacrylamide gel electrophoresis and transferred to a 0.45 μm nitrocellulose membrane. After blocking with 5% BSA in TBST, the membrane was incubated overnight at 4 °C with primary antibodies, including ZO-1 (1:2000; Abcam, Cambridge, UK), Occludin (1:1000; Abcam), Claudin-1 (1:1000; Abcam), TLR4 (1:1000; Abclonal, China), IL-1β (1:1000; CST, USA), MyD88 (1:1000; Proteintech, China), and β-actin (1:2000; Abcam). The membrane was then washed three times with TBST and incubated with horseradish peroxidase-conjugated secondary antibody for 90 min at room temperature. Finally, the membrane was washed with TBST, treated with ECL reagent (Solarbio), and the bands were captured and analyzed using a ChemiDoc XRS system (Bio-Rad).

### Immunofluorescence staining

For immunofluorescence microscopy, the colonic section was fixed with 4% paraformaldehyde and dehydrated through graded ethanol solutions [28]. The colonic section was permeabilized with PBS containing 0.1% (v/v) Tween 20, washed with an immunostaining washing solution (Beyotime Biotechnology, China), and blocked for 1 h with an immunostaining blocking solution. The colonic section was then incubated with primary antibodies overnight at 4°C. The primary antibodies used were as follows: rabbit anti-Mucin 2 (1:50; Proteintech, China), rabbit anti-ZO-1 (1:50; Proteintech, China), rabbit polyclonal anti-Claudin 1 (1:50; Proteintech, China), and rabbit polyclonal anti-Occludin (1:50; Proteintech, China). After incubation, the colonic section was washed three times with the washing solution and then incubated with secondary antibodies. The cell nuclei were stained with DAPI. Images were captured using fluorescence microscopy (Eclipse Ti-E, Nikon, Japan).

### RNA isolation and gene expression

Colon tissue was isolated from mice and stored at −80°C until further analysis. Colon tissues were homogenized with beads in Trizol (Sangon, China) reagent, and then reverse transcribed to cDNA using Evo M-MLV RT Premix for qPCR (AG11706, Accurate Biotechnology) with the manufacturer’s instructions. Quantitative real-time PCR was performed with a SYBR^®^ Green Premix qPCR Kit (AG11701, Accurate Biotechnology) on a Bio-Rad iQ5 PCR system (Bio-Rad, Hercules, CA, USA). All reactions were amplified in 45 cycles and The cycling conditions included 95°C for 30 s, 40 cycles of 95°C for 5 s, 55°C for 30 s and dissociation steps of 95°C for 15 s and 60°C for 30 s. The relative expression of the endogenous control gene, β-actin, was determined using the 2^−ΔΔCT^method [27]. The primers used in this assay were obtained from Shanghai Biotechnology and listed in Table 1.

**Table 1:** Gene and primer sequences

Genes	Forward	Reverse
*Muc2*	GCTGACGAGTGGTTGGTGAATG	GATGAGGTGGCAGACAGGAGAC
*ZO-1*	GACCTTGAGCAGCCGTCATA	CCGTAGGCGATGGTCATAGTT
*Occludin*	CAGCCTTCTGCTTCATCG	GTCGGGTTCACTCCCATTA
*Claudin-1*	CACTCCCAGACTCCACCACC	CGATCCATCCCAGAGAAGCC
*NF-κB*	ATGGCAGACGATGATCCCTAC	CGGAATCGAAATCCCCTCTGTT
*Il-1β*	GAAATGCCACCTTTTGACAGTG	CTGGATGCTCTCATCAGGACA
*MyD88*	GCTGGAACAGACCAACTAT	TCCTTGCTTTGCAGGTAAT
*Tlr4*	ATTTTACACCATATTGCCGTCT	CCTTGCATTCCTTTGGCGAGA

### SCFAs analysis

SCFAs mediate the function of immune cells to promote homeostasis in the body [34]. Feces stored at −80°C were thawed, weighed, acidified with 20 µL of dilute sulfuric acid (50%, v/v), 1000 µL of ether, and 1 µL of 2-ethylbutyric acid, and homogenized by vortexing and shaking for 3 min. Sonication was carried out in ice-cold water for 30 min, followed by incubation at −20°C for 30 min, and then centrifuged at 12,000 rpm for 15 min. The supernatant was transferred to a centrifuge tube containing 250 mg of anhydrous sodium sulfate, mixed thoroughly, and centrifuged at 12,000 rpm for 15 min. The resulting supernatant was then filtered through a 0.22 μm organic membrane (Millipore, USA). SCFAs in feces were quantified by gas chromatography-mass spectrometry (GC-MS, Agilent, Japan) using the following temperature parameters: 260°C, 1 µL injection volume, 50:1 split ratio, and a solvent delay of 2.5 min. The heating program was as follows: initial temperature 80°C, ramped at 40°C/min to 120°C, then at 10°C/min to 200°C, and finally held at 230°C for 2 min. Mass spectrometry was performed using an electron ionization (EI) source with the following conditions: ion source temperature 230°C, quadrupole temperature 150°C, transmission line temperature 250°C, and electron energy set to 70 eV. The mass spectrometer was operated in full scan mode (SCAN) with a mass range of m/z 30–300. SCFAs quantification in feces was determined using a standard curve.

### 16 S rRNA gene sequencing analysis

Mouse feces were collected on the last day of the intervention and total bacterial DNA was isolated from mouse fecal samples with the E.Z.N.A.^®^ soil DNA Kit (Omega Bio-Tek, U.S.). The microorganisms were amplified by PCR using primers 338 F 5’-ACTCCTACGGGGGAGGCAGCAG-3’ and 806R 5’-GGACTACHVGGGTWTCTAAT-3’. The highly variable V3-V4 region of the microbial 16 S rRNA gene was amplified by PCR. Sequencing was performed using the Illumina MiSeq platform (Majorbio Bio-pharm, Shanghai, China), and closed reference operational taxonomic units (OTUs) were selected with 97% sequence similarity from the study database (version 7.1 http://drive5.com/uparse/) and aligned to the SILVA128/16S bacterial database for taxonomic information. Sequencing data were analyzed on the Majorbio I-Sanger Cloud Platform free online platform (http://www.majorbio.com/) [28].

### RAW 264.7 cell culture

Thaw frozen RAW 264.7 macrophages at 37°C in a water bath. Following thawing, centrifuge the cells at 8,000 r/min for 5 min [28]. Discard the supernatant and resuspend the cell pellet in 2 mL of complete DMEM, which includes 20% fetal bovine serum, 1% non-essential amino acids, and 1% penicillin/streptomycin solution. Incubate the cells at 37°C with 5% CO_2_ for 24 h until the cells are 80–90% full grown. For subculture, treat the cells with 0.25% trypsin-EDTA and quench the digestion with DMEM. Centrifuge at 8,000 r/min for 5 min, discard the trypsin solution, and resuspend the cells in 2 mL of DMEM. Gently pipette to ensure cell suspension before transferring to culture dishes or plates as per the experimental design.

### Caco-2 cell culture and treatments

Caco-2 cells were typically cultured in DMEM supplemented with 10% fetal bovine serum (FBS) at 37°C and 5% CO_2_. Transwell inserts were employed for the Caco-2 cell permeability assay. Caco-2 cells were seeded in the upper chamber of a 24-well Transwell insert with a polycarbonate filter membrane (polyester, 0.4 μm pores, Corning, New York, NY, USA) and cultured for 21 days until the monolayer with a TEER value of 800–1000 Ω to form a tightly connected structure [45]. Cells were treated with FP, pFP and CFS for 24 h. A single-enriched *L. monocytogenes* suspension (2 × 10^7^ CFU/mL, 500 µL) was then added to the upper chamber, while fresh FBS-free DMEM (800 µL) was added to the basal chamber. After 3 h of incubation, the invasive *L. monocytogenes* in the basal chamber were enumerated on LB agar plates. Trans-epithelial electrical resistance (TEER) was measured using a Millicell ERS-2 instrument (Millipore, Burlington, MA, USA).

### Statistical analysis

Data are presented as means ± standard deviation. A one-way analysis of variance (ANOVA) was used to compare results among multiple groups, followed by Tukey’s post-hoc test for pairwise comparisons. Statistical analysis was conducted using GraphPad Prism 7.0, with a *p*-value of < 0.05 considered statistically significant. The normality of data distribution was assessed using the Shapiro-Wilk test, and homogeneity of variance was verified using Levene’s test before performing ANOVA. For microbiome-related analyses, multiple comparisons were made by Kruskal-Wallis test and were corrected using the Benjamini-Hochberg false discovery rate (FDR) procedure.

## Results

### FP, pFP and CFS caused no changes of cytokines, organ index and weight before the infection

**Fig. 1:**
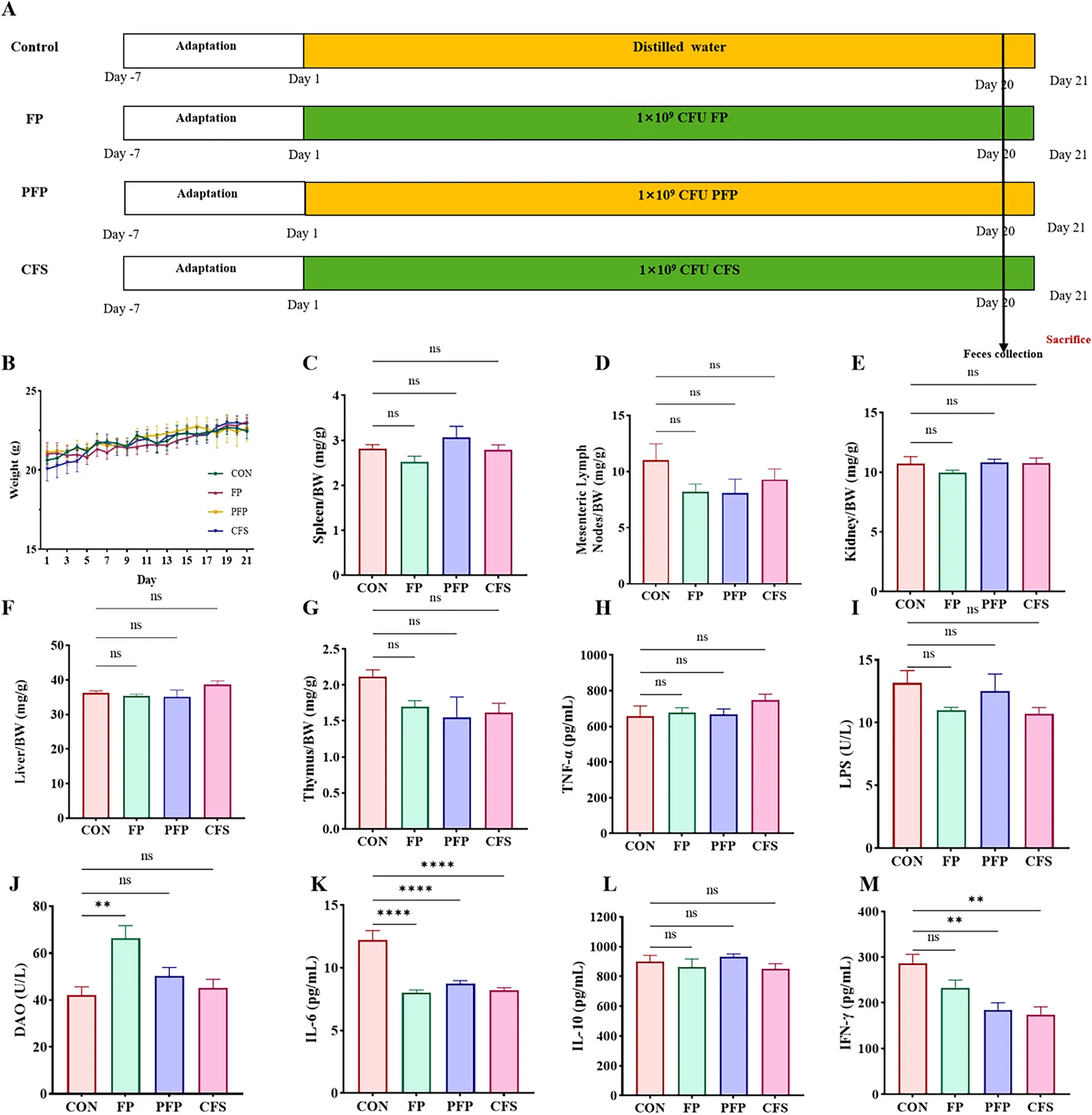
Effect of FP, pFP, and CFS on body weight, organ indices, and inflammatory factor in mice. **A** Experimental design. **B** Body weight. **C** Spleen weight. **D** Mesenteric Lymph Node weight. **E** Kidney weight. **F** Liver weight. **G** Thymus weight. **H** TNF-α. **I** LPS. **J** DAO. **K** IL-6. **L** IL-10. **M** IFN-γ. Data are expressed as mean ± SD. *p < 0.05, **p < 0.01, ***p < 0.001, ****p < 0.0001

The changes in body weight of mice over 21 consecutive days of gavage are shown in Fig. 1B. Over the first 6 days, no significant differences in body weight were observed among the con, pFP, and CFS groups, all of which showed an increase in body weight. No significant differences were observed in the liver, spleen, kidney, thymus, or mesenteric organ indices between the FP and con groups, or between the pFP and CFS groups (Fig. 1C-G). These results suggest that the FP, pFP, and CFS groups had no adverse effects on body weight or organ indices in mice.

To assess the effects of FP, pFP, and CFS interventions on systemic inflammation in mice, serum levels of inflammatory factors were measured using ELISA after 21 days of treatment. As shown in Fig. 1H-M, there were no significant differences in the levels of TNF-α, LPS, and IL-10 between the four groups. However, DAO levels were significantly higher in the FP group compared to the pFP, CFS, and con groups (*p* < 0.05). Although DAO levels were significantly higher in the FP group, this elevation did not coincide with increased inflammatory markers or histopathological damage, suggesting a physiological adaptation rather than an adverse effect. Additionally, FP, pFP, and CFS interventions significantly reduced IL-6 levels, and pFP and CFS also decreased IFN-γ levels. These results suggest that under normal conditions, FP, pFP, and CFS do not adversely affect body weight, organ indices, and inflammatory responses in mice, but reduce the levels of certain proinflammatory factors.

#### Effect of FP, pFP and CFS on colon, ileum and SCFAs production before the infection

**Fig. 2:**
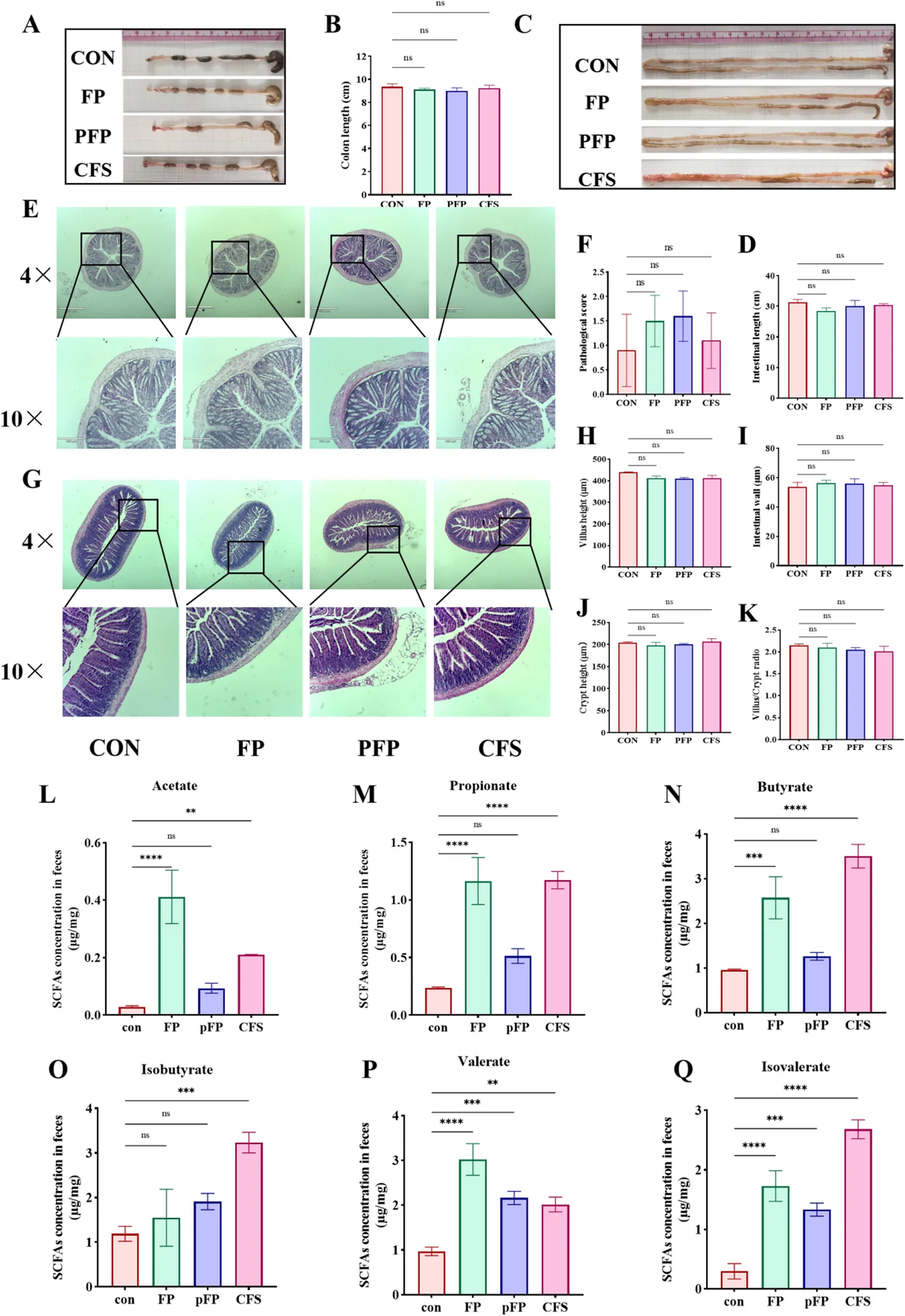
FP, pFP, and CFS treatments affect gut health in pre-infected mice. **A** Colon. **B** Colon length. **C** Ileum. **D** Ileum length. **E** Representative colon H&E staining image. Magnification (upper, × 40) (lower, × 100). **F** Histological scores. **G** Representative ileum H&E staining image. Magnification (upper, × 40) (lower, × 100). **H** Villus height. (**I**) Intestinal wall. **J** Crypt height. **K** Villus/Crypt radio. **L** Acetate. **M** Propionate. **N** Butyrate. **O** Isobutyrate. **P** Valerate. **Q** Isovalerate. Data are expressed as mean ± SD. (*p < 0.05, **p < 0.01, ***p < 0.001, ****p < 0.0001)

As shown in Fig. 2, the colon length was 9.36 ± 0.25 cm in the control group, 9.10 ± 0.14 cm in the pFP group, 9.00 ± 0.32 cm in the FP group, and 9.24 ± 0.29 cm in the CFS group, with no significant differences observed among the groups (*p* > 0.05). The ileum lengths were 31.48 ± 0.97 cm in the control group, 30.09 ± 1.87 cm in the pFP group, 28.56 ± 0.94 cm in the FP group, and 30.46 ± 0.44 cm in the CFS group (*p* > 0.05). H&E staining of the ileum and colon indicated that the sections from all four groups appeared normal, with no pathological differences observed (Fig. 2E, G).

As shown in Fig. 2L-Q, after 21 days of intervention, butyrate levels were 0.95 ± 0.021 µg/mg in the con group, 2.57 ± 0.47 µg/mg in the FP group, 1.26 ± 0.086 µg/mg in the pFP group, and 3.51 ± 0.26 µg/mg in the CFS group. The FP and CFS groups exhibited significantly higher levels than the con group (*p* < 0.05). The levels of valerate were 0.97 ± 0.0095 µg/mg in the con group, 3.02 ± 0.35 µg/mg in the FP group, 2.16 ± 0.15 µg/mg in the pFP group, and 2.01 ± 0.16 µg/mg in the CFS group, all significantly higher in the FP, pFP, and CFS groups compared to the con group (*p* < 0.05). Propionate levels were 0.24 ± 0.0059 µg/mg in the con group, 0.51 ± 0.064 µg/mg in the pFP group, 1.16 ± 0.20 µg/mg in the FP group, and 1.17 ± 0.075 µg/mg in the CFS group, with significant differences observed between the con group and the FP, pFP, and CFS groups. Isovalerate levels were 0.30 ± 0.13 µg/mg in the con group, 1.33 ± 0.11 µg/mg in the pFP group, 1.73 ± 0.26 µg/mg in the FP group, and 2.68 ± 0.16 µg/mg in the CFS group. Additionally, propionate, isobutyrate and isovalerate levels were higher in the feces of mice in the CFS group, while acetic and propionate levels were higher in the FP group. These results indicate that FP, pFP, and CFS interventions increased the production of SCFAs.

#### Pretreatment with FP, pFP or CFS modulates *L. monocytogenes* infection

**Fig. 3:**
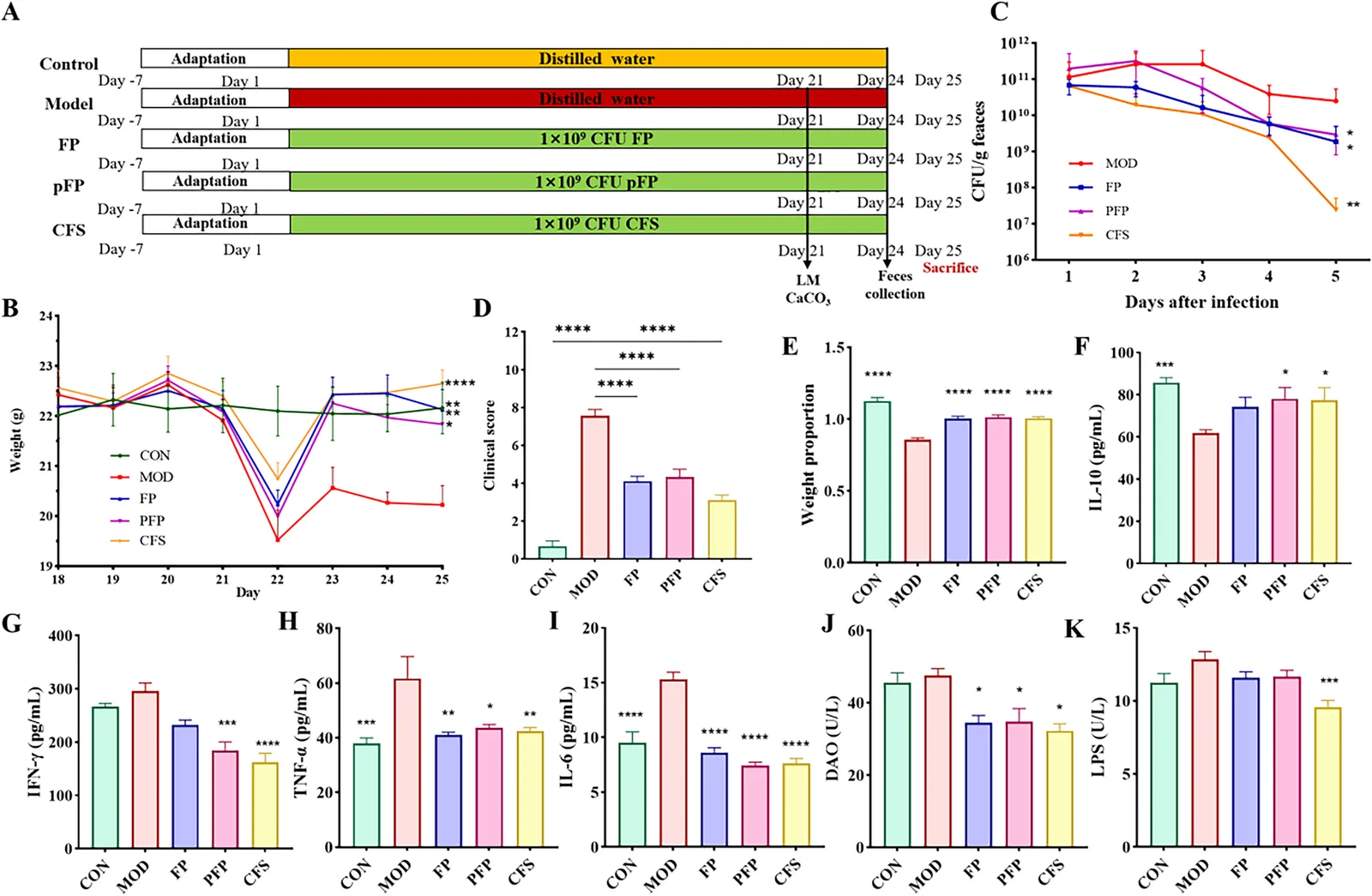
FP, pFP, and CFS treatment attenuated *L. monocytogenes* infection. **A** Experimental design. **B** Body weight changes. **C** *L. monocytogenes* in feces. **D** Clinical score. **E** Weight proportion. The concentrations of the inflammatory cytokines. **F** IL-10. (G) IFN-γ. (H) TNF-α. **I** IL-6. **J** DAO. (K) LPS. Data are expressed as mean ± SD. (*p < 0.05, **p < 0.01, ***p < 0.001, ****p < 0.0001)

To assess the effects of FP, pFP, and CFS on mice during infection, we tested mice body weight and fecal colony counts (Fig. 3B). The body weight of mice treated with FP, pFP, and CFS decreased gradually during the infection, while the infected group continued to lose weight on day 2. In contrast, the body weight of FP, pFP, and CFS-treated mice returned to baseline levels. *L. monocytogenes* was assessed using the plate counting method, and mice pretreated with FP, pFP, and CFS consistently had lower fecal bacterial loads than the mod group throughout the course of infection (Fig. 3C, *p* < 0.05). These results suggest that FP, pFP, and CFS can delay weight loss and reduce fecal bacterial load in *L. monocytogenes*-infected mice, indicating their potential efficacy in preventing infection.

Serum levels of inflammatory factors reflect the severity of systemic *L. monocytogenes* infection. Higher bacterial loads trigger greater secretion of inflammatory factors by the host in response to *L. monocytogenes* invasion. To assess the level of systemic inflammation, we measured the expression of inflammatory factors in the serum of infected mice using ELISA. As shown in Fig. 3F-K, serum levels of five inflammatory factors (IL-6, DAO, LPS, TNF-α, and IFN-γ) were significant change (*p* < 0.05). The secretion of all five inflammatory factors was significantly higher in the mod group than in the FP, pFP, and CFS-treated groups. IL-10 levels were significantly higher in the FP, pFP, and CFS-treated mice compared to the mod group.

### FP, pFP and CFS reduce bacterial counts in feces and organs of mice infected with *L. monocytogenes*

**Fig. 4:**
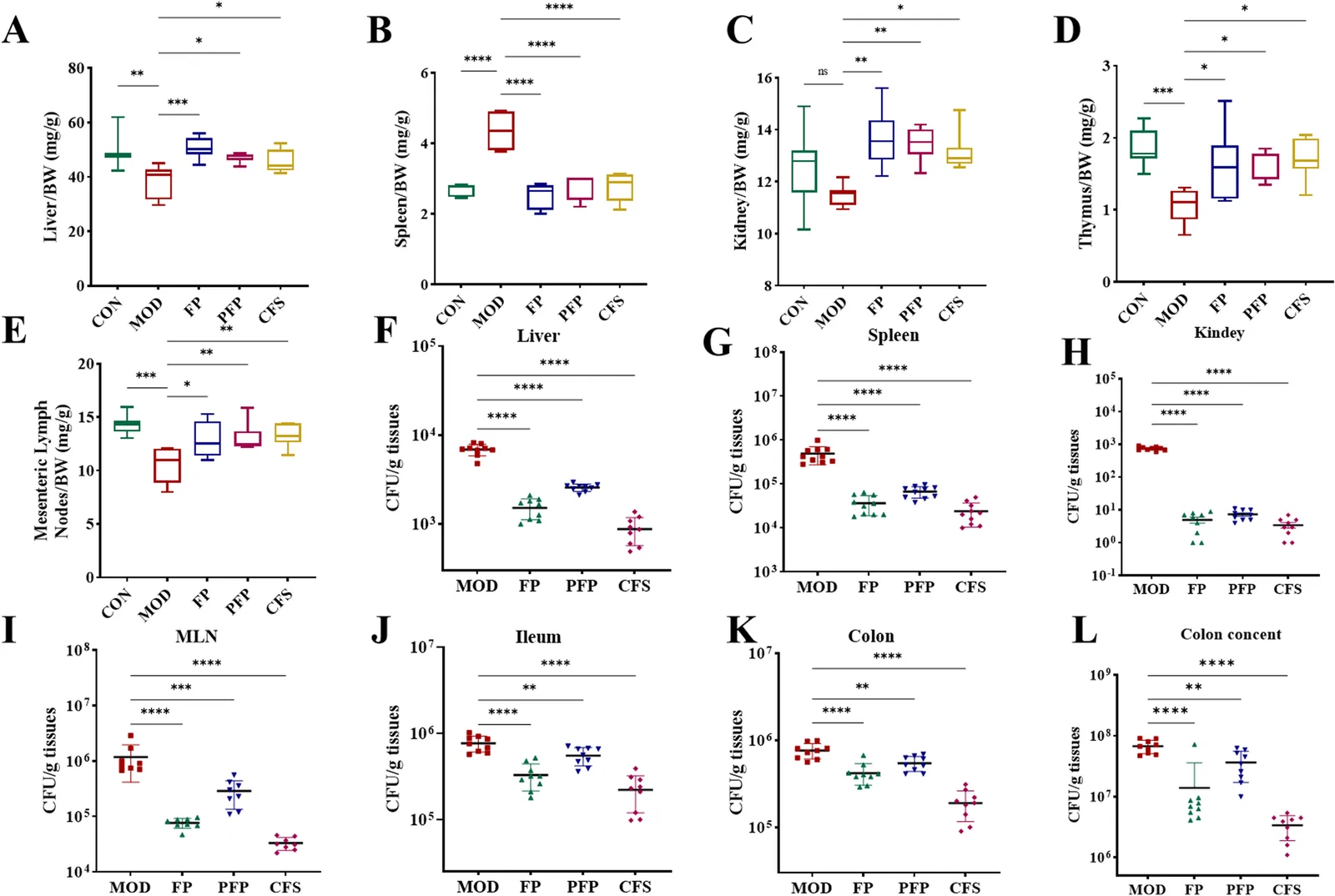
FP, pFP, and CFS reduced the number of bacteria in the feces and organs of mice infected with *L. monocytogenes.* Weight of multiple organs. **A** Liver. **B** Spleen. **C** Kidney. **D** Thymus. **E** MLN. **F** Liver bacterial load. **G** Spleen bacterial load. **H** Kidney bacterial load. **I** MLN bacterial load. **J** Ileum bacterial load. **K** Colon bacterial load. **L** Colon content bacterial load. Data are expressed as mean ± SD. (*p < 0.05, **p < 0.01, ***p < 0.001, ****p < 0.0001)

On day 3 post-infection, gross examination of the organs from infected mice revealed reduced splenomegaly in FP, pFP, and CFS-treated groups (*p* < 0.0001). However, the liver, kidney, thymus, pancreas, and mesenteric indices did not differ significantly between the five groups compared to the control group but were significantly lower than those in the mod group (Fig. 4). *L. monocytogenes* load was significantly reduced in extraintestinal tissues, including spleen, liver, kidney, MLN, colon, and ileum, in mice pretreated with FP, pFP, and CFS after infection.

#### FP, pFP, and CFS reduce intestinal inflammation in *L. monocytogenes*-infected mice

**Fig. 5:**
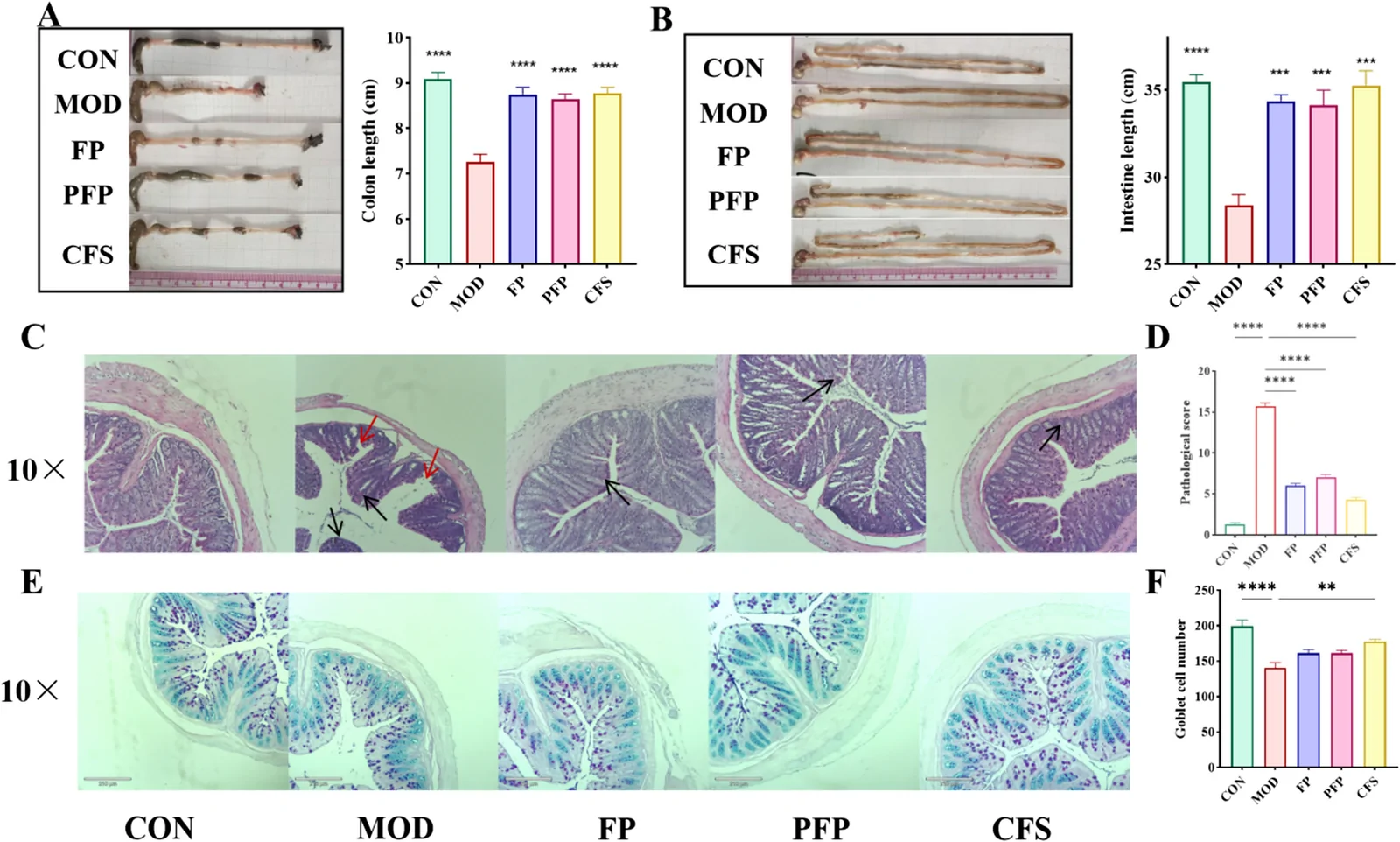
FP, pFP, and CFS reduce intestinal inflammation and tissue damage in *L. monocytogenes*-infected mice. **A** Colon. **B** Ileum. **C** Representative H&E staining image. Magnification (upper: 40 ×, lower: 100 ×, Black arrow: inflammatory cell infiltration; Red arrow: epithelial cell loss). **D** Histological scores. **E** Representative AB/PAS staining image. **F** Goblet number. Data are expressed as mean ± SD. (*p < 0.05, **p < 0.01, ***p < 0.001, ****p < 0.0001)

After infection, the colon length was measured as follows: 8.79 ± 0.63 cm in the control group, 7.81 ± 0.76 cm in the mod group, 8.83 ± 0.56 cm in the FP group, 8.59 ± 0.62 cm in the pFP group, and 8.80 ± 0.63 cm in the CFS group. These results suggest that FP, pFP, and CFS interventions alleviated the shortening of colon length in mice. Colon tissue pathology was further analyzed by H&E and AB/PAS staining (Fig. 5C-F). Compared to the FP, pFP, and CFS-treated mice, the model mice exhibited more severe pathological damage, characterized by a notable reduction in goblet cells, infiltration of numerous inflammatory cells, and disruption of intestinal epithelial integrity (*p* < 0.01). These results indicate that FP, pFP, and CFS interventions enhance host resistance to *L. monocytogenes*-induced enteritis. These findings demonstrate that FP, pFP, and CFS can reduced *L. monocytogenes* load in tissues, and attenuate colon tissue damage, highlighting their beneficial role in the host’s defense against systemic *L. monocytogenes* infections.

#### FP, pFP, and CFS reduce *L. monocytogenes*-induced intestinal barrier damage

**Fig. 6:**
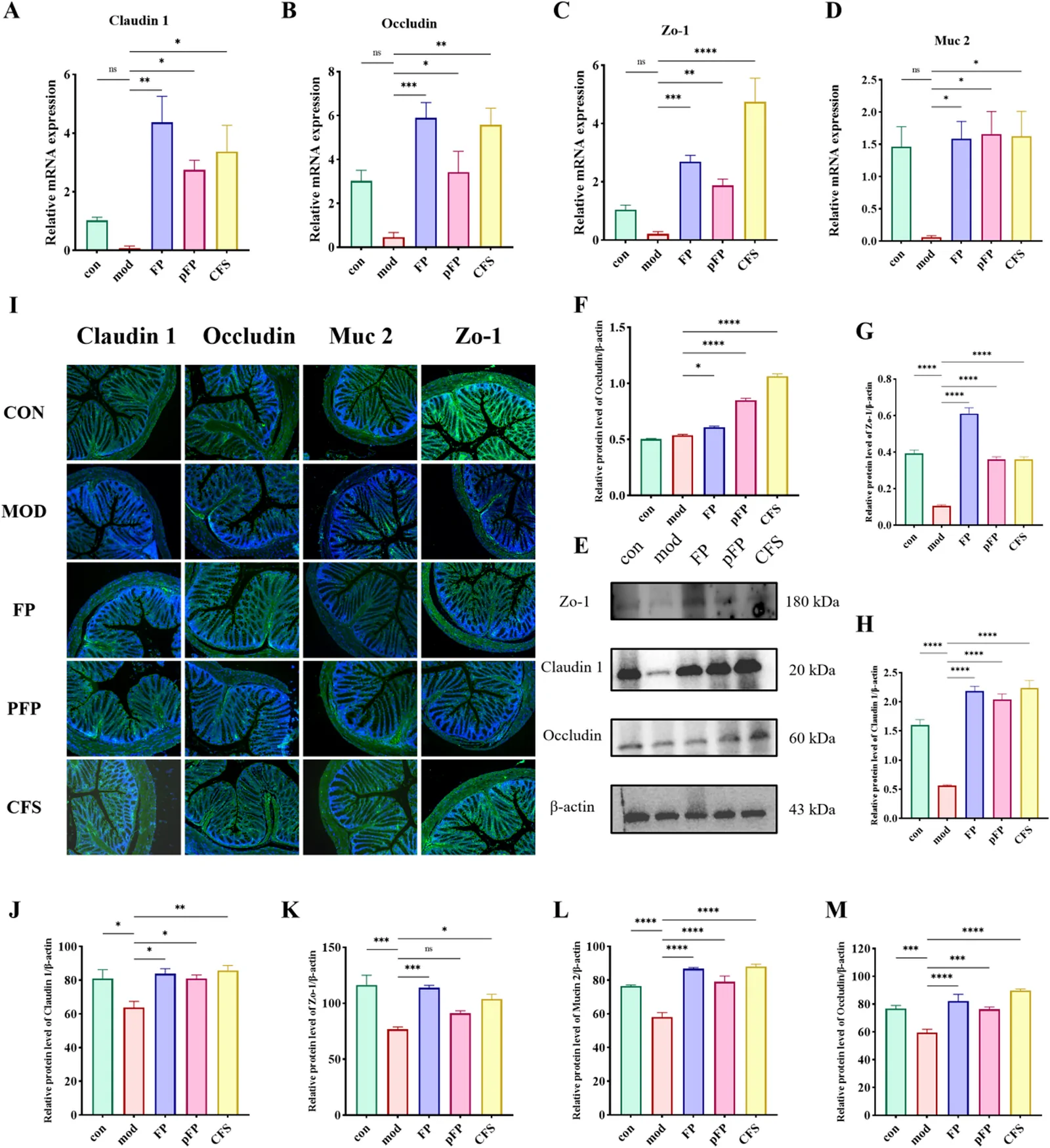
Effect of FP, pFP and CFS on tight junction protein expression in *L. monocytogenes*-infected mice. **A** mRNA expression of *Claudin 1*. **B** mRNA expression of *Occludin*. **C** mRNA expression of *ZO-1*. **D** mRNA expression of *Muc2*. **E** Western blots for tight junction proteins. **F** Quantification of Claudin 1. **G** Quantification of Occludin. **H** Quantification of ZO-1. **I** Immunofluorescence images of tight junction proteins. **J** Quantification of Claudin 1 (IF). **K** Quantification of Occludin (IF). **L** Quantification of ZO-1 (IF). **M** Quantification of Muc2 (IF). Data are expressed as mean ± SD. (*p < 0.05, **p < 0.01, ***p < 0.001, ****p < 0.0001)

As shown in Fig. 6A-D, RT-qPCR analysis revealed a significant upregulation of *Claudin 1*, *Occludin*, *ZO-1*, and *Mucin 2* gene expression in the FP, pFP, and CFS groups (*p* < 0.05). To further investigate the expression of intestinal barrier-related proteins, Western blot analysis was performed for Claudin 1, Occludin, and ZO-1. The results showed a significant increase in the expression of these proteins in the FP, pFP, and CFS groups compared to the mod group (Fig. 6E-H). Immunofluorescence staining of intestinal tissues (Fig. 6I) also confirmed FP and CFS upregulation of protein expression.

#### FP, pFP, and CFS downregulate the TLR4/MyD88/NF-κB signaling pathway

**Fig. 7:**
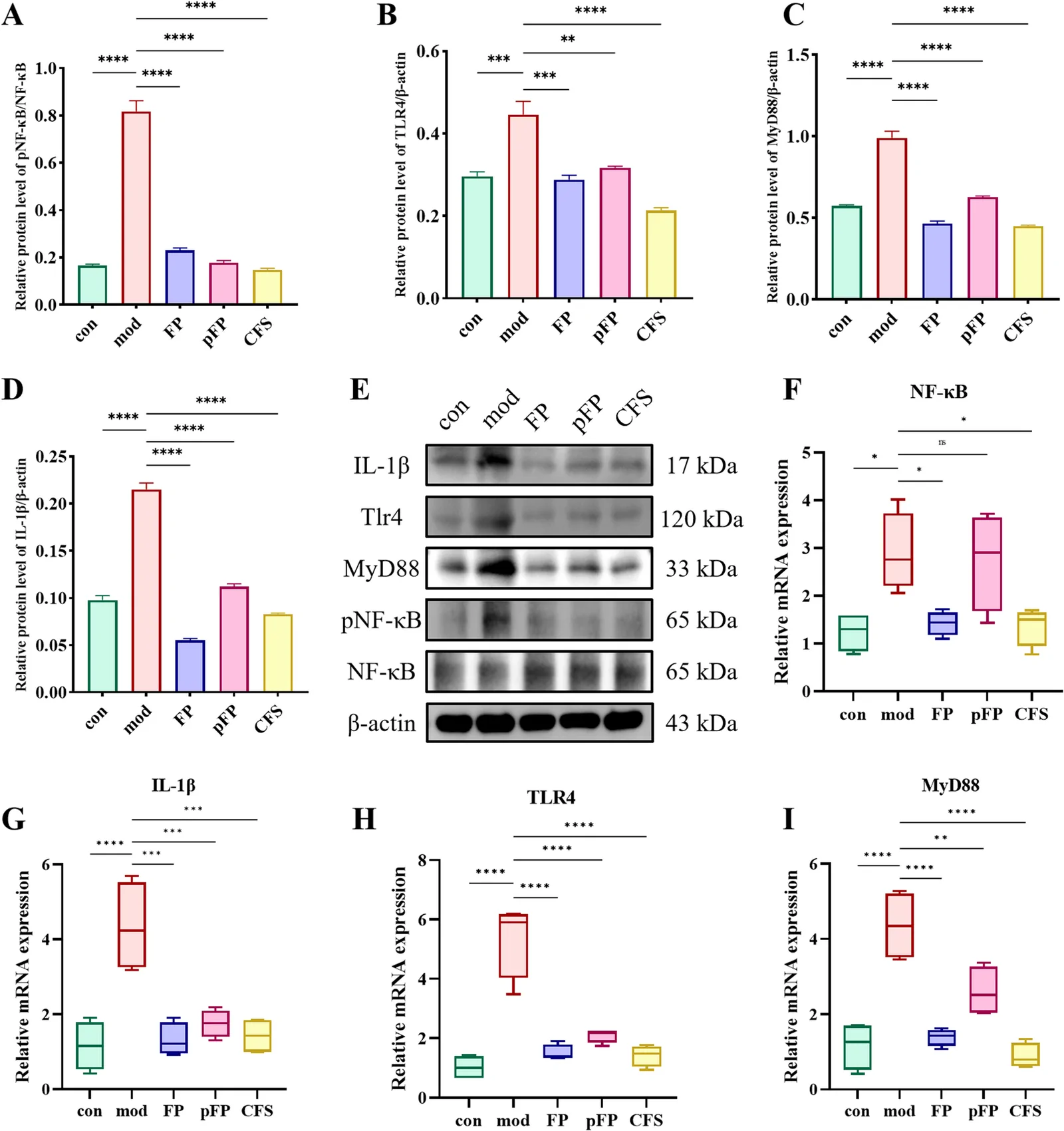
Effect of FP, pFP and CFS on MyD88 protein expression and TLR4/NF-κB pathway in infected mice. **A** Western blot of NF-κB. **B** Western blot of TLR4. **C** Western blot of MyD88. **D** Western blot of IL-1β. **E** Western blots for TLR4/NF-κB pathway. **F** mRNA expression of *NF-κB*. **G** mRNA expression of *IL-1β*. **H** mRNA expression of *TLR4*. **I** mRNA expression of *MyD88*. (*p < 0.05, **p < 0.01, ***p < 0.001, ****p < 0.0001)

To investigate the potential role of FP, pFP, and CFS in activating inflammatory pathways during *L. monocytogenes* infection, we assessed the gene and protein expression of MyD88 and the downstream cytokine IL-1β (Fig. 7). RT-qPCR analysis revealed significant downregulation of NF-κB, TLR4, MyD88, and IL-1βgene expression in the colon of FP, pFP, and CFS-treated mice (*p* < 0.05). These findings were further validated by Western blot, showing significant downregulation of the corresponding proteins (Fig. 7E and F). These results suggest that the protective effects of FP, pFP, and CFS are associated with the downregulation of the TLR4/NF-κB signaling pathway and MyD88 expression.

#### FP, pFP and CFS ameliorated intestinal microbial dysregulation caused by *L. monocytogenes* infection

**Fig. 8:**
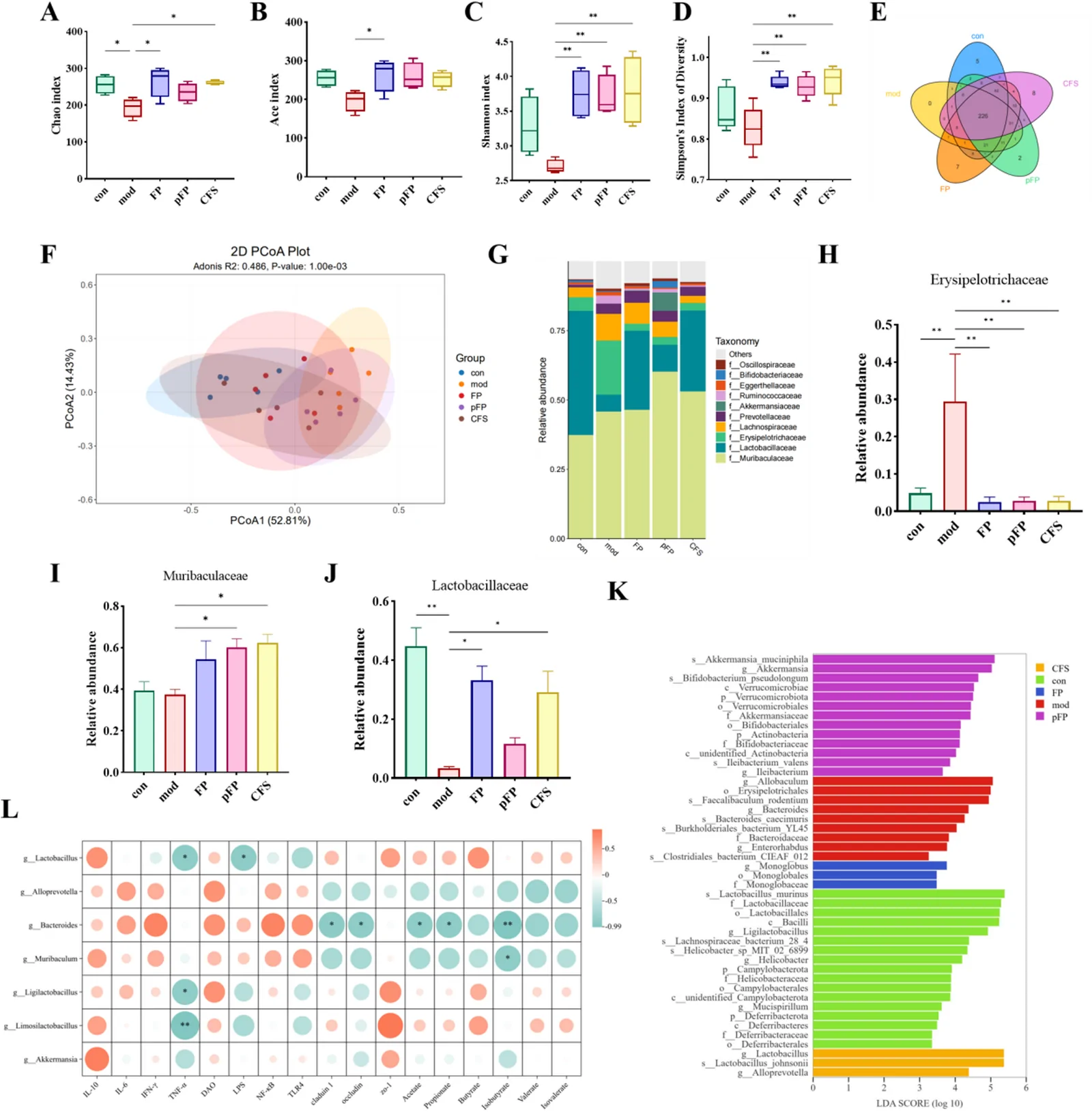
FP, pFP and CFS alleviate gut microbial dysbiosis induced by *L. monocytogenes* infection. **A** Chao index. **B** Ace index. **C** Shannon index. **D** Simpson’s Index of Diversity. **E** Venn diagram of shared and unique bacteria at the OTU level. **F** PCoA plot analysis. **G** Comparison of gut microbiota composition at the family level. **H** Relative abundance of Erysipelotrichaceae. **I** Relative abundance of Muribaculaceae. **J** Relative abundance of Lactobacillaceae. **K** LEfSe analysis LDA scores. **L** Spearman correlation analysis between gut microbiota and gut inflammation-related index (*p < 0.05, **p < 0.01, ***p < 0.001)

The gut microbiota is one of the key factors in regulating host health. The gut microbial diversity and composition changes in feces after FP, pFP and CFS intervention were analyzed by 16 S rRNA gene sequencing. As evidenced in Fig. 8A-D, the alpha-diversity analysis was used to evaluate evaluated community richness (Chao index, Ace index) and diversity (Shannon index, Simpson’s index of Diversity). Compared to the control group mice, the FP group and CFS group showed a higher Simpson’s index of Diversity, whereas the LM group exhibited a significant decrease in the Simpson’s index of Diversity. The results of the experiment revealed a noteworthy shift in the microbial community due to *L. monocytogenes* infection, in comparison to the control groups. Figure 8E displays the Venn diagram at the OTU level for each of the five groups. The control group exhibited 356 OTUs, the LM group had 337, the FP group had 390, the pFP group had 375 and the CFS group had 377 OTUs, indicating no significant alteration in the number of bacterial species. Subsequently, to further characterize the intestinal microbiota between the five groups, principal coordinate analysis (PCoA) revealed aggregation between samples from the same group of mice in the con, mod, FP, pFP, and CFS groups, and the CFS group maintained a similar microbiota to the con majority after infection. Figure 8G-J show the relative abundance of family in mice. Among them, the mod group showed a significant increase in the abundance of Erysipelotrichaceae and Ruminococcaceae and a significant decrease in the abundance of Lactobacillaceae compared to the other four groups. Probiotics Muribaculaceae also increased in the intestinal microbiota after FP, pFP and CFS interventions. To further investigate the differences in microbial composition among the experimental groups, we employed LEfSe with a log LDA score cutoff of > 3.0 and *p*-value < 0.05 to identify differentially expressed bacteria. Histograms of the relative abundance of various bacteria are presented in Fig. 8K. It was observed that CFS group manifested higher levels of Lactobacillus. On the other hand, the mod group displayed higher relative abundance levels of Erysipelotrichales. The pFP group demonstrated a greater abundance of *Akkermansia muciniphila*. In Fig. 8L, this heatmap illustrates the correlation between 7 gut bacterial genera and inflammation-related factors, gut barrier-related factors, as well as SCFAs. The correlations between specific taxa and inflammatory markers suggest a potential link or association, rather than definitive evidence that these bacteria directly modulate the inflammatory response. Specifically, Lactobacillus showed significant positive correlations with IL-10, Zo-1, and butyrate. Bacteroides exhibited strong positive correlations with TNF-α and DAO, but significant negative correlations with occludin and SCFAs such as acetate. Akkermansia was mainly strongly positively correlated with IL-10. Alloprevotella was negatively correlated with multiple SCFAs. Muribaculum showed a significant negative correlation with isobutyrate. Ligilactobacillus was positively correlated with butyrate. Limosilactobacillus showed a strong positive correlation with acetate. These correlations suggest that different gut bacterial genera may be associated with host physiological states, potentially through their relationships with immune responses, affecting gut barrier function, and altering SCFAs levels, with Bacteroides and Lactobacillus playing important roles in immune regulation and metabolite production.

#### Effects of FP, pFP and CFS on SCFAs after infection

**Fig. 9:**
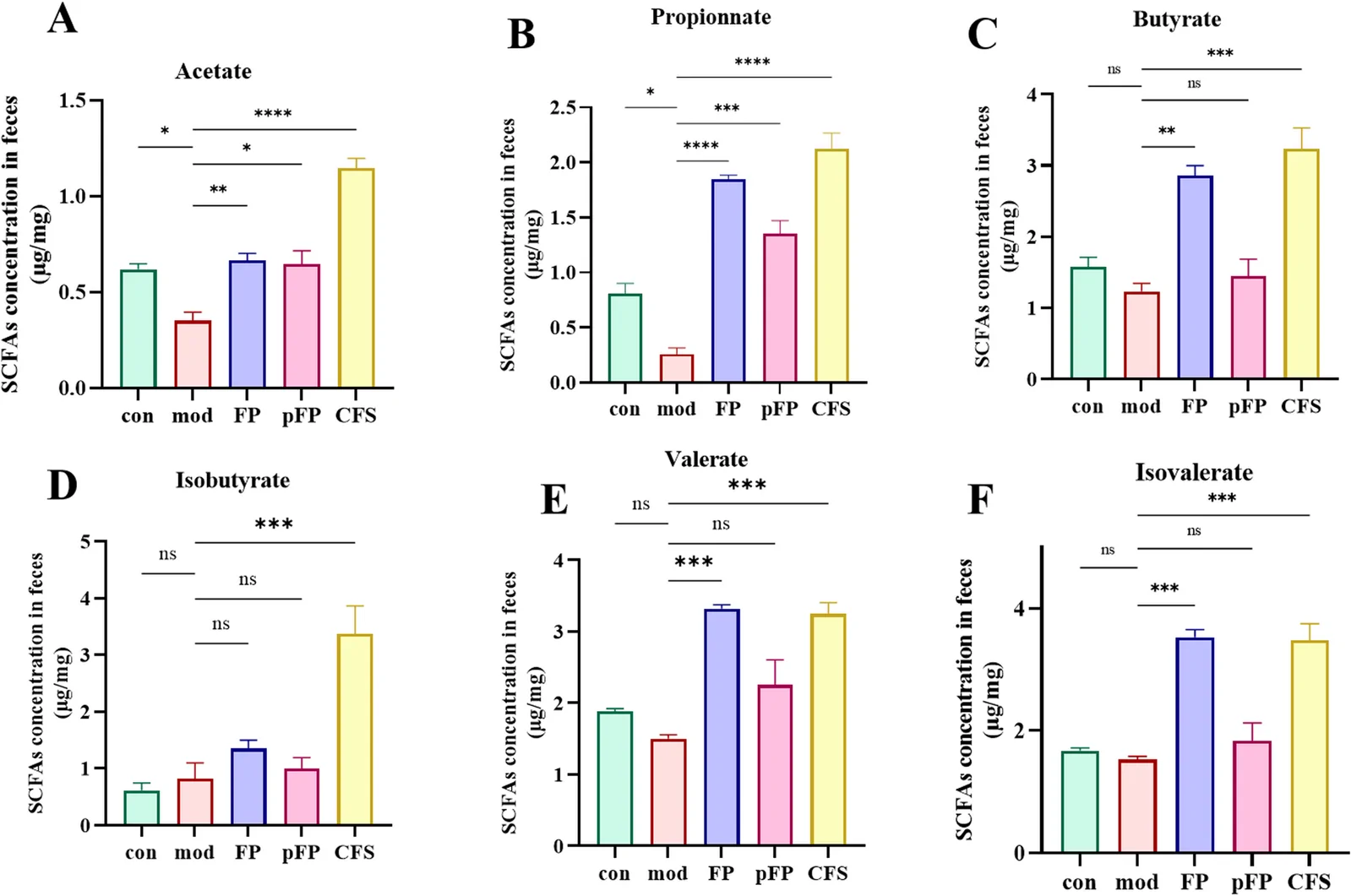
Effect of FP, pFP and CFS on SCFAs in *L. monocytogenes*-infected mice. **A** Acetate (**B**) Propionate (**C**) Butyrate (**D**) Isobutyrate (**E**) Valerate and (**F**) Isovalerate were determined by GC-MS in different treatment groups. Data are expressed as mean ± SD. (*p < 0.05, **p < 0.01, ***p < 0.001, ****p < 0.0001)

SCFAs not only serve as an energy source for intestinal epithelial cells but also provide colonization resistance to the host. This underscores the importance of SCFAs for host health. Therefore, we next analyzed the levels of SCFAs in the feces of mice infected with *L. monocytogenes* after the 21 days intervention. As shown in Fig. 9, no significant difference in SCFAs levels was observed between the post-infection control and pFP groups. However, the propionate content in the FP and CFS groups was 1.85 ± 0.058 µg/mg and 2.12 ± 0.25 µg/mg, respectively, while butyrate levels were 2.86 ± 0.24 µg/mg and 3.23 ± 0.51 µg/mg, and valerate levels were 3.32 ± 0.091 µg/mg and 3.24 ± 0.28 µg/mg. These values were significantly higher than those in the other two groups (*p* < 0.05). These results indicate that FP and CFS positively regulate the production of propionate, butyrate and valerate after infection. This may be one of the mechanisms through which FP and CFS reduce the tissue load of *L. monocytogenes* and alleviate infection.

### FP, pFP and CFS affected inflammatory factor levels in Raw 264.7 cells and slowed down intestinal barrier damage

**Fig. 10:**
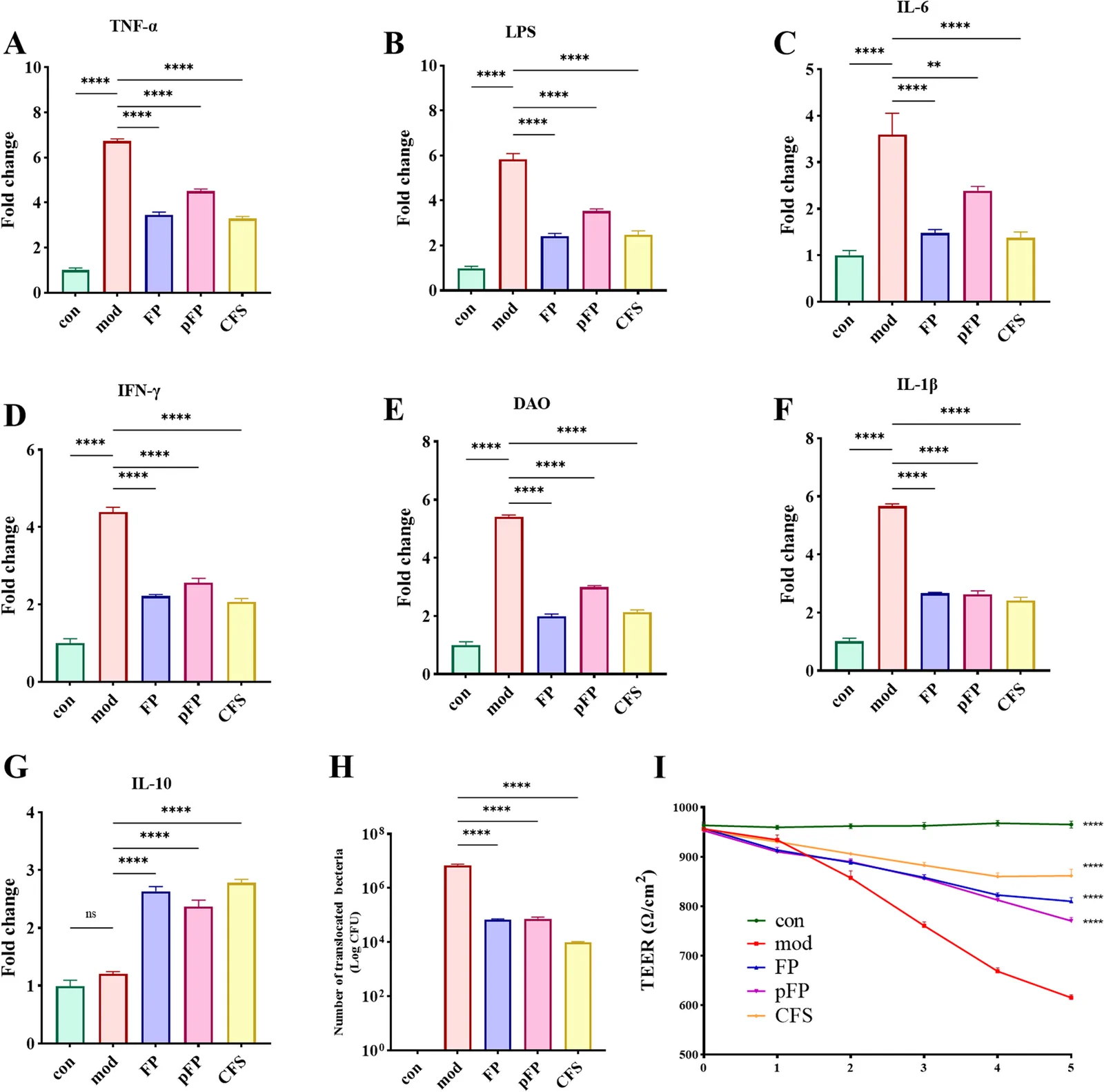
Effect of FP, pFP and CFS on inflammatory factors and permeability of Raw 264.7 cells and Caco-2 cells. **A** TNF-α. **B** LPS. **C** IL-6. **D** IFN-γ. **E** DAO. **F** IL-1β. **G** IL-10. **H** Bacterial translocation content. **I** TEER values for Caco-2 cell monolayers. Data are expressed as mean ± SD. (*p < 0.05, **p < 0.01, ***p < 0.001, ****p < 0.0001)

To investigate the effects of FP, pFP, and CFS on inflammatory factor production in Raw 264.7 cells (Fig. 10). The intervention groups significantly suppressed pro-inflammatory factor levels and increased the anti-inflammatory factor IL-10 compared to the mod group.

An in vitro intestinal epithelial barrier model was developed using Caco-2 cells. FP, pFP, and CFS pretreatment reduced bacterial translocation of *L. monocytogenes* through the Caco-2 monolayer (*p* < 0.05) (Fig. 10H). TEER changes, expressed as TEER%, are shown in Fig. 10I, with values significantly lower in the mod group compared to the FP, pFP, and CFS groups. The trans-epithelial resistance permeability assay further demonstrated that FP, pFP, and CFS attenuated the loss of barrier function induced by *L. monocytogenes*. In conclusion, FP, pFP, and CFS may mitigate *L. monocytogenes* infection by enhancing the intestinal epithelial barrier.

## Discussion

*F. prausnitzii*is widely used as a probiotic, offering potential benefits in regulating intestinal microecological balance, enhancing host immunity, and inhibiting harmful bacterial growth. It also aids in preventing and treating intestinal infections, improving nutrient digestion and absorption, reducing intestinal inflammation, and may have positive effects on cardiovascular health and metabolic diseases [23, 35]. However, the role of *F. prausnitzii* in the context of *L. monocytogenes* infection remains poorly understood. This study investigates the role of *F. prausnitzii* in the course of *L. monocytogenes* infection. Our findings indicate that live *F. prausnitzii*, pasteurized *F. prausnitzii*, and its cell-free supernatant significantly attenuate intestinal damage and disease severity caused by *L. monocytogenes* infection in mice via down-regulation of TLR4/NF-κB signaling pathway expression.

Regarding the baseline biosafety, our results showed that FP, pFP, and CFS interventions exerted no adverse effects on body weight, organ indices, or intestinal histomorphology, consistent with the recognized safety profile of *F. prausnitzii *[36]. Notably while major systemic inflammatory markers remained stable the significant reduction in IL-6 and IFN-γ levels suggests a potential immunomodulatory effect under physiological conditions. This aligns with recent findings that probiotic strains can modulate host immunity even in the absence of disease states indicating that these interventions may establish a protective immunological baseline prior to pathogenic challenge [1, 20].

The intestinal barrier is an important line of defense for the body in which the mucus layer and epithelial cells in the intestines form a physical barrier against potentially toxic substances that play a vital role in maintaining intestinal health [4]. Histopathologic analyses revealed severely impaired epithelial cell integrity in *L. monocytogenes*-infected mice whereas the FP pFP and CFS groups showed improved pathological features including increase goblet cells improved epithelial cell structure and reduced inflammatory cell infiltration and mucosal damage. Previous studies have reported that histological analyses of infections with foodborne pathogens usually show a lack of goblet cells in the colonic epithelium [51]. Tight junction proteins are membrane-associated proteins that regulate material flow between cells, which tightly connects adjacent cells and establishes a barrier [55]. During intestinal inflammation, the expression and distribution of ZO-1 may be altered, disrupting tight junctions and increasing intestinal barrier permeability [14]. Impaired Occludin function leads to relaxation of tight junctions, potentially causing abnormal release of cytokines such as tumor necrosis factor-alpha (TNF-α) and interleukin-6 (IL-6), which further promote inflammation [22, 26]. Mucin-deficient mice exhibit increased susceptibility to intestinal infections, indicating that the mucus layer plays a critical role in the defense against intestinal pathogens [5, 16]. *F. prausnitzii *restored intestinal barrier function and ameliorated metabolic disorders, while promoting Mucin2 secretion from goblet cells in DSS-induced colitis. In FP, pFP, and CFS-treated mice, high expression of Mucin2 and other tight junction proteins were observed after infection. This observation aligns with the known role of FP in protecting the gut by remodeling the mucus layer and stimulating mucus production by goblet cells. These findings support the hypothesis that FP, pFP, and CFS may prevent infection, in part, by enhancing markers of intestinal barrier function. However, we recognize that functional validation of barrier permeability in vivo is limited in the current study, as direct permeability assays were not performed. Therefore, future studies will be conducted to investigate this aspect more thoroughly. We plan to perform in vivo permeability assays, such as FITC-dextran flux measurements, to provide direct functional evidence of barrier protection. Furthermore, the mechanistic insights from our study could be further elucidated by integrating multi-omics approaches. For instance, studies utilized integrated transcriptomic and metabolomic analyses to investigate probiotic-mediated host responses during bacterial infections [19, 43]. Their work demonstrated how probiotics can modulate host immunity, metabolism, and intestinal barrier integrity, providing a framework for understanding the broader systemic effects observed in our intervention. Future work combining our phenotypic data with such multi-omics technologies could reveal novel pathways through which FP, pFP, and CFS confer protection against enteric pathogens.

MyD88 (myeloid differentiation factor 88) is an intracellular signaling molecule, ubiquitously expressed in immune cells, and serves as a key adaptor for Toll-like receptors (TLRs) [6, 15]. As a critical signaling adapter, MyD88 bridges the NF-κB/TLR4 pathways [13, 38]. This leads to NF-κB activation and nuclear translocation, initiating an immune response. Proinflammatory cytokines increase the permeability of intestinal tight junction proteins. In the present study, we found that FP, pFP and CFS reduced the expression of the intracellular adaptor protein MyD88 and downregulated the pro-inflammatory cytokine IL-1β in colon tissues. This is consistent with what previous studies have found, *Lactobacillus rhamnosus*GG (LGG) helps maintain intestinal barrier integrity and reduces the passage of pathogens and harmful substances, thereby diminishing NF-κB pathway activation [42]. Similar to NF-κB, TLR4 is responsible for the production of pro-inflammatory cytokines. TLR4 acts as a pattern recognition receptor that recognizes pathogen-associated molecular patterns such as bacterial lipopolysaccharides (LPS), which in turn activates downstream signaling pathways and contributes to inflammatory responses. During the signaling process of TLR4, connector proteins such as MyD88 and TRIF play a key role by recruiting and activating the IKK complex, which ultimately leads to the activation of NF-κB. Similarly, epigallocatechin gallate (EGCG) from green tea and proanthocyanidins from grape seed extract, both with anti-inflammatory and antioxidant properties, modulate the TLR4/NF-κB pathways [58]. Similarly, treatment with FP, pFP, and CFS downregulated MyD88 protein, modulating the TLR4/NF-κB pathway and reducing intestinal damage. In our study, the downregulation of TLR4, MyD88, and NF-κB pathway components suggests a potential involvement of this signaling axis in the protective effects of FP, pFP, and CFS. However, it is important to note that these findings are based on expression changes, which provide associative evidence rather than direct mechanistic proof. Further in-depth investigations, such as employing TLR4 knockout models or specific pathway inhibitors, will be conducted in our future work to definitively elucidate the precise molecular mechanisms.

SCFAs in the intestines are mainly derived from the fermentation process of dietary fiber by intestinal microorganisms [57]. These SCFAs have a variety of important roles in the gut such as providing energy to intestinal cells regulating the immune system and inhibiting the growth of harmful bacteria as well as having a positive effect on the prevention of intestinal inflammation improvement of the intestinal barrier function and reduction of cardiovascular disease risk [49]. There is growing evidence that the levels of SCFAs play an important role in the intestinal barrier and inflammation. Among these is the demonstration that butyrate acid provides energy to intestinal cells through oxidative metabolism and promotes cell proliferation and repair, which is essential for maintaining the integrity of the intestinal barrier [50]. Furthermore, SCFAs are able to regulate the expression and distribution of tight junction proteins. Studies have shown that butyrate increases the expression of tight junction proteins such as occludin and compactin, thereby enhancing the sealing of the intestinal barrier and reducing intestinal permeability [31]. In the present study, FP, pFP and CFS interventions restored the decrease in fecal SCFAs content induced by *L. monocytogenes *induction. In particular, FP and CFS significantly increased the content of SCFAs, mainly propionate, butyrate and valerate. It has been reported that propionate deficiency may lead to decreased expression of tight junction proteins, increased intestinal permeability, and an imbalance in intestinal microbiota, which together increase intestinal permeability to pathogens and harmful substances [33]. Meanwhile, significant changes in alpha diversity and microbial composition were observed in the intestinal tracts of mice in the mod group compared to the control group and Bacteroidetes were relatively enriched after *L. monocytogenes *infection. This is the same as the results of the previous study which showed significant differences in the alpha diversity indices Richness index and Chao1 index between fecal samples of the IBD group and Treat group and Mannose oligosaccharides (MOS) can promote the diversity of gut microbiota including Akkermansia and Bacteroides species and inhibit certain pathogens [9]. However, Lactobacillus were enriched even more after FP, pFP and CFS interventions. This is in line with previous research that probiotics, such as *F. prausnitzii*, *Bifidobacterium* and *Lactobacillus*, can help prevent or reduce dysbiosis [47]. Thus, our findings suggest that FP, pFP and CFS treatment is associated with mitigated *L. monocytogenes* infection and a better maintained gut microbiota composition and increased intestinal inflammation.

*F. prausnitzii*is a well-known butyrate producer, and this SCFA is central to its anti-inflammatory and barrier-strengthening effects by modulating immune responses, inhibiting histone deacetylases (HDACs), and serving as a primary energy source for colonocytes [7]. Additionally, the secretome of *F. prausnitzii* is rich in bioactive molecules, such as microbial anti-inflammatory molecules (MAMs) that maintain intestinal integrity, as well as bacteriocins, enzymes, and signaling peptides with immunomodulatory properties [52]. Beyond these, the CFS represents a complex matrix of metabolic products that may include other organic acids, vitamins, extracellular polysaccharides (EPS), and fatty acid amides, all of which have been implicated in host-microbe communication and immune regulation [37, 48]. Nevertheless, we explicitly acknowledge a significant limitation of the current study regarding the absence of comprehensive metabolomic and proteomic characterization of the CFS. Future investigations will be conducted to deeply analyze the specific bioactive components and their underlying mechanisms.

## Conclusion

In conclusion, our study demonstrates that live *F. prausnitzii*, pasteurized *F. prausnitzii*, and its cell-free supernatant effectively attenuate *L. monocytogenes* infection in mice. The protective mechanisms are associated with the enhancement of intestinal barrier integrity, the restoration of gut microbiota homeostasis, the replenishment of SCFAs, and are also correlated with downregulation of the TLR4/MyD88/NF-κB signaling pathway, corresponding to the observed reduction in inflammatory responses. The limitations of this study include the lack of an in vivo permeability assays and incomplete identification of the specific bioactive components responsible for these effects. Future research employing integrated metabolomic and proteomic analyses is warranted to isolate the key functional molecules and further elucidate the precise mechanisms underlying the postbiotic properties of *F. prausnitzii*.

## Data Availability

The main data supporting the findings are included in the article. Data are available for research purposes on reasonable request.
